# Early and long-term skull growth after surgical correction for sagittal synostosis in relation to the occurrence of papilledema

**DOI:** 10.1007/s00381-022-05629-x

**Published:** 2022-09-02

**Authors:** Stephanie D. C. van de Beeten, Melissa S. I. C. Kurniawan, Nathalie W. Kamst, Sjoukje E. Loudon, Irene M. J. Mathijssen, Marie-Lise C. van Veelen

**Affiliations:** 1 Department of Plastic and Reconstructive Surgery and Hand Surgery, Erasmus Medical Center, Room Ee15.91, 2040, 3000 CA Rotterdam, the Netherlands; 2 Department of Ophthalmology, Erasmus Medical Center, Rotterdam, the Netherlands; 3 Department of Neurosurgery, Erasmus Medical Center, Rotterdam, the Netherlands

**Keywords:** Sagittal synostosis, Intracranial hypertension, Papilledema, Craniofacial, Head circumference

## Abstract

**Objective:**

Stagnation of skull growth is correlated with papilledema in craniosynostosis. In this retrospective cohort study, we describe the postoperative skull growth after surgical correction for sagittal synostosis and its relation to the development of papilledema.

**Methods:**

Patients with isolated sagittal synostosis at our center between 2005 and 2012 were included. Occipitofrontal circumference (OFC) was analyzed, at 3 time points (preoperative, 2 years postoperative, and last OFC measurement) and 3 phases (initial postoperative growth, long-term growth, and overall growth), and related to papilledema on fundoscopy.

**Results:**

In total, 163 patients were included. The first time interval showed a decline in skull growth, with subsequent stabilization at long term. Papilledema occurred postoperatively in 10 patients. In these patients, the OFC at 2 years and at last follow-up (T3) were significantly smaller than in patients without papilledema. A larger OFC resulted in a decreased odds of developing papilledema at both postoperative time points (at T2 (OR = 0.40,* p* = 0.01) and at T3 (OR 0.29, *p* < 0.001)). Sensitivity and specificity analysis indicated that an OFC below 0.25 SD at T2 (sensitivity 90%, specificity 65%) and below 0.49 at T3 (sensitivity 100%, specificity 60%) are related to the occurrence of papilledema.

**Conclusion:**

A small OFC is correlated with the occurrence of papilledema. A decline in OFC within 2 years postoperatively is common in sagittal synostosis and is acceptable up to a value of 0.25SD. Patients with an OFC at last follow-up of less than 0.5SD are at risk for developing papilledema.

## Introduction

Early synostosis of the sagittal suture results in scaphocephaly which is characterized by an elongated skull, bulging forehead, and prominent occiput. Surgery is performed before the age of 1 year to prevent intracranial hypertension (ICH) and to restore cosmesis. However, 2–9% develops ICH postoperatively [1–5]. The development of ICH during follow-up in isolated sagittal synostosis patients seems to be related to reduced intracranial volume (ICV) in particular [6]. A stagnation of the skull growth has been correlated to papilledema, an indirect sign of ICH, in trigonocephaly [7]. Although growth curves of trigonocephaly and unicoronal patients are similar, the growth curve of sagittal synostosis patients seems to follow a different growth pattern [1]. Patients with sagittal synostosis typically present with a large occipitofrontal head circumference (OFC) of around + 2 SD. After surgery, a decrease of OFC curve within the first 2 years is observed [1, 3, 5, 6]. This decline finally stabilizes at an OFC that remains larger compared to the normal population. Similar results are found in studies describing long-term follow-up after various operation techniques [8, 9]. It is unknown which decline in skull growth results in an increased risk of raised intracranial pressure. Therefore, our first aim is to describe the postoperative and long-term growth of the OFC after surgical correction and relate impaired skull growth to the development of papilledema. Our second aim is to derive cutoff points of the OFC during follow-up for the risk of developing papilledema based on our population.

## Methods

### Study population

This retrospective cohort study was performed at the Dutch Craniofacial Center (Sophia’s Hospital, Erasmus University Medical Center, Rotterdam). All patients diagnosed with isolated sagittal synostosis who were operated on between 2005 and 2012 were included. Exclusion criteria were syndromic patients, surgery performed elsewhere, and patients with an incomplete follow-up (< 3 years after surgery or no preoperative OFC was excluded). The study protocol followed the statements of the Declaration of Helsinki.

### Treatment algorithm

At our center, the treatment of sagittal synostosis patients involves frontobiparietal remodeling (FBR), extended strip craniotomy (ESC), and spring-assisted correction (SAC), depending on the age and year of presentation. Between 2005 and 2010, patients presenting before the age of 6 months underwent ESC. Patients presenting after the age of 6 months underwent FBR. After 2010, the protocol for early surgery changed; patients presenting before the age of 6 months underwent SAC.

Patient data was collected prospectively according to our treatment protocol including fundoscopy and OFC at regular intervals preoperative, at intake, and during subsequent examination. Postoperatively, fundoscopy and OFC were obtained at 3 months post-surgery, at the age of 1, 2, 4, and 6 years. After the age of 6, the OFC measurements continue every 3 years until the age of 18 years. When ICH was suspected based on the appearance of repeated papilledema on fundoscopy, skull growth arrest, or clinical signs, such as frequent headache, an additional CT or MRI was obtained and invasive intracranial pressure monitoring was considered. Invasive ICP monitoring was obtained for 24 h and evaluated according to the following criteria: baseline ICP value during the day and overnight (< 10 mmHg, normal; 10–15 mmHg, borderline; and > 15 mmHg, abnormal). In addition, values at the beginning and end of the night were compared to check for any overnight increase in ICP. Plateau waves were evaluated based on height (< 25 mmHg, normal; 25–35 mmHg, borderline; > 35 mmHg, abnormal) and duration (< 10 min, normal; 10–20 min, borderline; > 20 min, abnormal). In case of high pressures during ICP, a surgical revision will be performed.

### Fundoscopy

Patients were screened for ICH through fundoscopy by an experienced ophthalmologist. Mydriasis was achieved with phenylephrine tropicamide. Patients were dichotomized for the presence of papilledema. After two positive funduscopic examinations, papilledema was scored as “yes.”

Papilledema on fundoscopy needs to be differentiated from pseudopapilledema. Pseudopapilledema is defined as anomalous elevation of the optic disks without edema of the fiber layer, caused by, for example, hyperopic crowded nerve or optic disk drusen [10, 11]. In contrast to papilledema, pseudopapilledema is not caused by ICH. Papilledema and pseudopapilledema can co-exist. Patients with solely pseudopapilledema were stratified in the no papilledema group. To confirm drusen, a B-scan ultrasound (Aviso, Quantel Medical, Clermont-Ferrand, France) was performed. Patients suspected of hyperopia underwent refractive error measurement, 30 min after instillation of cyclopentolate 1% using an autorefractor (Topcon, Tokyo Optical Co., Japan).

#### OFC

**Fig. 1:**
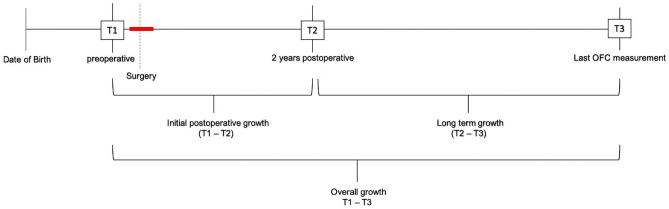
Timeline occipitofrontal circumference measurements. T1, preoperatively; T2, 2 years post-surgery; T3, last follow-up

OFC was measured by a trained nurse practitioner and represents the head circumference in an axial plane from the forehead to the most prominent point in the occiput. The OFC is measured in centimeters and translated into standard deviations based on the Dutch Standard OFC growth curve [12]. Therefore, OFC measurements of sagittal synostosis patients can be compared to the Dutch population. For study purposes, 3 time points were established to asses OFC values: T1 preoperatively, T2 2 years post-surgery, T3 last follow-up (Fig. 1). T2 was evaluated 2 years post-surgery, since recent studies report the decline in postoperative growth to stabilize after 2 years post-surgery [1, 3, 6]. T3, as last follow-up, is defined as the last measurement that has been reported at time of the data collection. This measurement needed to be at least 3 years postoperative, to observe the difference between the decline in the first 2 years postoperative and the stabilization of the OFC after 2 years.

Based on the time points, three phases of skull growth will be evaluated: initial postoperative growth (T1–T2), long-term growth (extending from 2 years post-surgery to last follow-up, T2–T3), and overall growth (T1–T3) (Fig. 1).

### Statistical analysis

The presence of papilledema was defined as a dichotomized variable. The OFC was measured over time and 3 time points were established. The baseline characteristics were defined with percentages for categorical variables, with mean and SD for normally distributed continuous variables, and with median and interquartile range (IQR) for non-normal distributed variables. The differences of mean OFC at the different time points and phases for patients with and without papilledema were tested with a Mann–Whitney *U* test. Results were adjusted for multiple testing. To look into the effect of developing papilledema in relation to the OFC at different time points, the odds ratio (OR) was calculated using an univariate logistics regression. The differences of the OFC between surgical techniques were tested with an ANOVA. When significant differences were found, Tukey’s test for post hoc analysis was performed. For all measurements with a significant effect on the presence of papilledema according to the Mann–Whitney *U* test and univariate analysis, the predictive value was determined. This was performed using a receiver operating characteristics (ROC) curve analysis. The sensitivity and specificity were calculated corresponding to the threshold determined on the Youden Index. For all ROC curves, the high area under the ROC curve (AUC) was determined to provide an aggregate measure of performance across all possible classification thresholds. Data were analyzed using RStudio Version 3.6.1., and *p* < 0.05 was considered significant.

## Results

In total, 258 patients with sagittal synostosis were operated between 2005 and 2012, of which 95 were excluded due to incomplete follow-up of OFC measurements and fundoscopy 2 years after surgery. Of the included 163 patients, the majority was male (82.2%). FBR was performed in 40 patients (24.5%), ESC in 91 (55.83%), and SAC in 32 (19.51%), with a mean age at T3 of 5.67 years (SD 1.82) and a median follow-up time of 5.18 years (95% CI: 5.08–5.63) (Table 1). In 8 patients, measurements at T2 were missing; therefore, the initial postoperative growth and the long-term growth could not be calculated. Of these 8 patients (1 FBR, 6 ESC, and 1 SAC), 1 patient had papilledema and 7 did not.

**Table 1:** Descriptives

	Overall (*n* = 163)
Gender	
Males	134 (82.21%)
Females	29 (17.79%)
Type of surgery	
FBR	40 (24.54%)
ESC	91 (55.82%)
SAC	32 (19.63%)
Age at surgery (years) (mean ± SD (95% CI))	
FBR	1.07 ± 0.61 (0.87–1.26)
ESC	0.42 ± 0.06 (0.41–0.43)
SAC	0.46 ± 0.05 (0.44–0.48)
Age at last follow-up measurement (years) (mean ± SD (95% CI))	
FBR	6.46 ± 1.77 (5.89–7.03)
ESC	5.95 ± 1.72 (5.59–6.31)
SAC	3.89 ± 0.72 (3.64–4.17)
Follow-up time (median (95% CI)) (years between preoperative measurement and postoperative measurement)	
FBR	5.47 (5.19–6.38)
ESC	5.79 (5.36–6.08)
SAC	3.69 (3.63–3.92)
Last	
Postoperative papilledema	10 (6.13%)
Reoperation due to papilledema	7 (4.29%)

*FBR* frontobiparietal remodeling, *ESC* extended strip craniotomy, *SAC* spring-assisted correction

### Fundoscopy

Preoperatively, 3 patients had papilledema (1 FBR, 1 ESC, 1 SAC). After surgery, papilledema resolved within the first 4 months.

Ten patients (6.13%) developed papilledema postoperatively. None of these 10 patients had preoperative papilledema. In addition, one patient was diagnosed with pseudopapilledema based on hyperopic crowded disk. The mean age at first postoperative observation of papilledema was 4.13 years (95% CI 3.27–4.98). The mean time period between the first positive and second positive fundoscopy is 2 months (95% CI 1.44–2.56). In these latter patients, 9 underwent ICP monitoring and one patient refused ICP monitoring. The mean time period between first positive fundoscopy and ICP measurement was 7.20 months (95% CI 2.03–12.4). Of these 9 patients, 5 had increased ICP, 3 had borderline, and one had normal ICP. Overall 7 patients were re-operated. After reoperation, papilledema resolved after 8.75 months (95% CI 2.42–15.1). In the patients who did not undergo surgery, papilledema resolved spontaneously after follow-up including repeated fundoscopies, with a mean period of 23.88 months (range 17.8–31.3).

In 9 patients, invasive ICP monitoring was conducted. Increased ICP was found in 5 patients. In 4 of these patients, surgical revision was performed, after which papilledema resolved. In one patient with increased ICP, papilledema disappeared during work-up for surgery, after which re-expansion was canceled. Papilledema did not reoccur. Borderline ICP was observed in 3 patients. In the first patient, papilledema was persistent and visual evoked potentials were abnormal; therefore, surgery was indicated and papilledema resolved postoperatively. The second patient with borderline ICP had symptoms of ICH including frequent headache and behavior changes, a vertex bulge, and a stable Chiari I malformation on MRI. Surgical revision was performed and resulted in a reduction of symptoms and resolved papilledema. The third patient with borderline ICP had initially a normal OFC and no clinical signs (headache complaints, etc.) and the MRI showed no signs of ICH. After watchful waiting, papilledema persisted and complaints progressed over time, after which surgical revision was performed. Papilledema resolved after surgery.

One patient revealed normal pressures during ICP monitoring and therefore, watchful waiting was maintained, after which papilledema resolved.

#### OFC

In 155 patients, a complete follow-up of OFC measurement at all time points was available. At T1, the mean OFC was 1.66 SD. After surgery, there was a decline in initial postoperative growth (mean difference T1 − T2 =  − 0.94). This decline stabilizes during long-term growth (mean difference T2 − T3 =  − 0.05) (Table 2).

**Table 2:** Mean occipitofrontal circumference between patients with papilledema and without papilledema, Mann–Whitney test

	No (*N* = 153)	Yes (*N* = 10)	Total (*N* = 163)	Mean difference	Conf. int	*p* value
T1				0.32	(− 0.31, 0.95)	0.411
Mean (SD)	1.68 (1.05)	1.36 (0.86)	1.66 (1.04)			
T2				0.82	(0.43, 1.21)	0.010
Mean (SD)	0.76 (1.06)	− 0.06 (0.48)	0.71 (1.05)			
T3				0.98	(0.53, 1.43)	0.002
Mean (SD)	0.75 (1.02)	− 0.23 (0.60)	0.69 (1.03)			
Initial postoperative growth (T1–T2)				0.68	(0.23, 1.12)	0.009
Mean (SD)	− 0.89 (0.87)	− 1.57 (0.60)	− 0.94 (0.87)			
Long-term growth (T2–T3)				− 0.03	(− 0.25, 0.20)	0.748
Mean (SD)	− 0.05 (0.53)	− 0.02 (0.29)	− 0.05 (0.52)			
Overall growth (T1–T3)				0.66	(0.20, 1.12)	0.016
Mean (SD)	− 0.93 (0.93)	− 1.59 (0.61)	− 0.97 (0.93)			

*T1* preoperatively, *T2* 2 years post-surgery, *T3* last follow-up

### OFC in patients with and without papilledema

**Fig. 2:**
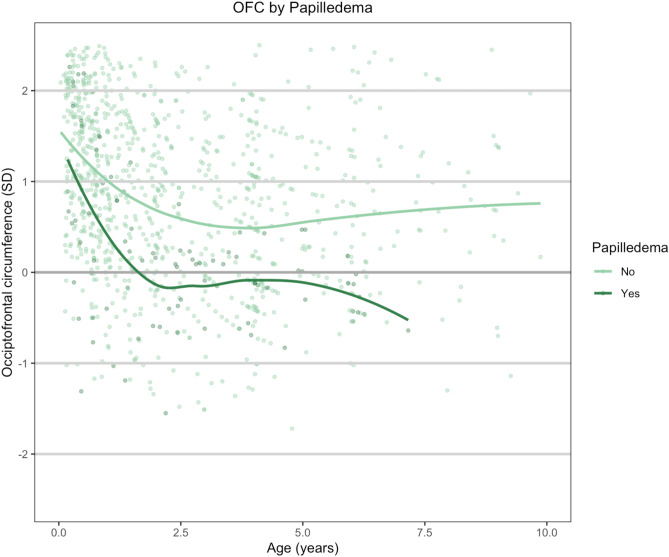
Occipitofrontal circumference over time for patients with and without papilledema

Figure 2 shows OFC at different time points and different time intervals for patients with and without papilledema. The mean preoperative OFC (T1) did not differ between patients with and without papilledema (Table 2).

OFC at both T2 and T3 was significantly smaller for patients with papilledema than patients without papilledema. At T2, the odds of developing papilledema is 60% lower in patients with a 1 SD larger OFC (OR = 0.40, 95% CI 0.18 to 0.90). At T3, the odds of developing papilledema is lower (OR = 0.29, 95% CI 0.12 to 0.68) in patients with an OFC of 1 SD larger (Table 3).

**Table 3:** Logistics regression for occipitofrontal circumference on papilledema

	OR	95% OR	*p* value (LR)
Papilledema (1/0)			
T1	0.74	(0.39, 1.39)	0.344
T2	0.40	(0.18, 0.90)	0.015
T3	0.29	(0.12, 0.68)	0.001
Initial postoperative growth (T1–T2)	0.31	(0.12, 0.83)	0.010
Long-term growth (T2–T3)	1.09	(0.30, 3.97)	0.892
Overall growth (T1–T3)	0.32	(0.13, 0.8)	0.008

*OR* odds ratio

With respect to the three phases of skull growth, the initial postoperative growth (T1–T2), and overall growth (T1–T3), a significantly larger decline was seen for the patients with papilledema (*p* = 0.010, *p* = 0.008 resp.).

### Sensitivity and specificity of OFC measurements to predict papilledema

**Fig. 3:**
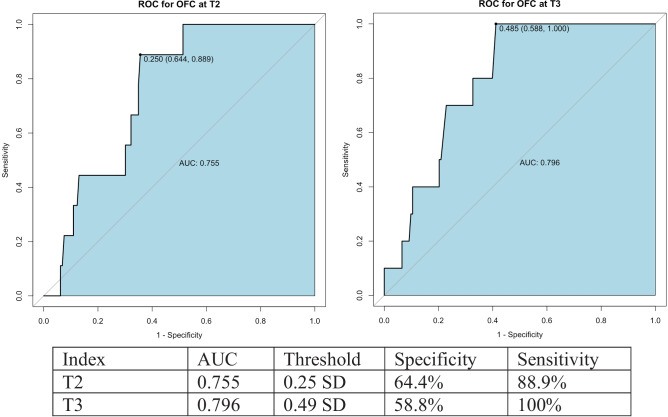
ROC curves, AUC, and threshold for OFC at T2 and T3. ROC, receiver operating characteristics curve; AUC, area under the ROC curve; T1, preoperatively; T2, 2 years post-surgery; T3, last follow-up

Predictive values for the OFC measurements at T2 and T3 were evaluated for the occurrence of papilledema (Fig. 2). Sensitivity analysis using OFC at T2 with a threshold of 0.25 SD yielded a best balance of specificity and sensitivity of 64.4% and 88.9%, respectively, for papilledema. This indicates that an OFC smaller than 0.25 SD is related to the occurrence of papilledema. At T3, the threshold is 0.49 with a specificity of 58.8% and sensitivity of 100% (Fig. 3).

### Type of surgery

**Fig. 4:**
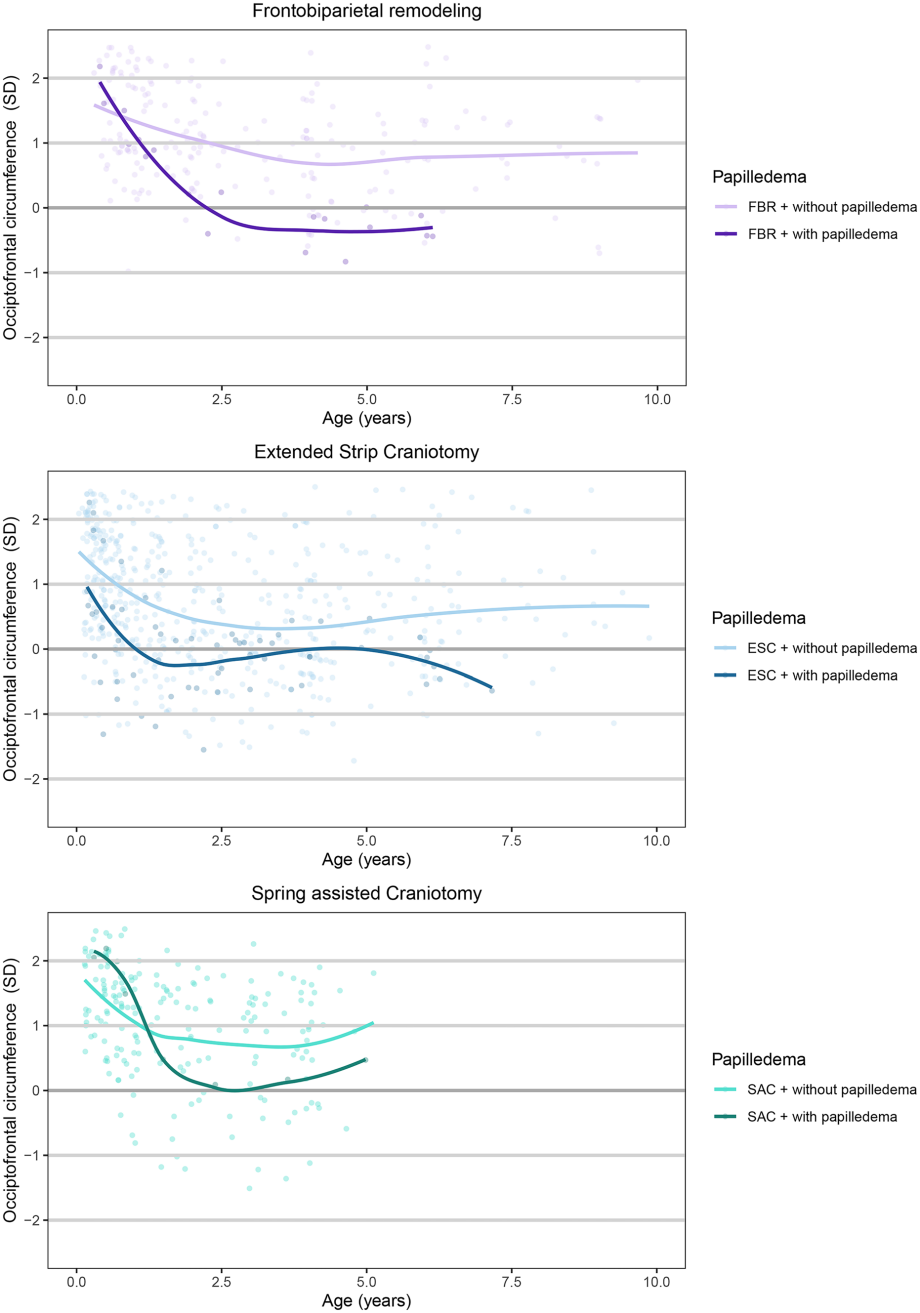
Mean occipitofrontal circumference over time for patients with and without papilledema per type of surgery. FBR, frontobiparietal remodeling; ESC, extended strip craniotomy; SAC spring-assisted correction; T1, preoperatively; T2, 2 years post-surgery; T3, last follow-up

Figure 4 shows the OFC for patients with papilledema and without papilledema, per type of surgery. No significant differences were found in OFC between surgical techniques at the different time points (Table 4). However, with respect to the phases of skull growth, the decline in initial postoperative growth (T1–T2) and long-term growth (T2–T3) was different. The decline in initial postoperative growth was smaller in patients who underwent FBR (− 0.56 ± 0.87) compared to patients who underwent ESC (− 1.07 ± 0.86) or SAC (− 1.10 ± 0.75) (*p* = 0.005), whereas the decline during long-term growth is larger for FBR (− 0.29 ± 0.57) compared to ESC (0.04 ± 0.54) or SAC (0.01 ± 0.27) (*p* = 0.003).

**Table 4:** Mean occipitofrontal circumference between surgical techniques

	FBR (*N* = 40)	ESC (*N* = 91)	SAC (*N* = 32)	Total (*N* = 163)	*p* value
T1					0.71
Mean (SD)	1.55 (0.93)	1.67 (1.17)	1.75 (0.77)	1.66 (1.04)	
T2					0.12
Mean (SD)	1.01 (0.96)	0.59 (1.11)	0.68 (0.94)	0.71 (1.05)	
T3					0.98
Mean (SD)	0.70 (0.94)	0.68 (1.12)	0.71 (0.88)	0.69 (1.03)	
Initial postoperative growth (T1–T2)					0.005
Mean (SD)	− 0.56 (0.87)	− 1.07 (0.86)	− 1.10 (0.75)	− 0.94 (0.87)	
Long-term growth (T2–T3)					0.003
Mean (SD)	− 0.29 (0.57)	0.04 (0.54)	0.01 (0.27)	− 0.05 (0.52)	
Overall growth (T1–T3)					0.64
Mean (SD)	− 0.85 (0.92)	− 1.00 (0.94)	− 1.04 (0.74)	− 0.97 (0.93)	

*FBR* frontobiparietal remodeling, *ESC* extended strip craniotomy, *SAC* spring-assisted correction, *T1* preoperatively, *T2* 2 years post-surgery, *T3* last follow-up

## Discussion

In this study, we described the postoperative skull growth at short- and long-term growth after surgical correction in patients with sagittal synostosis. Furthermore, we examined the relation between impaired skull growth and the development of papilledema.

Three core findings arise from this study. First, the OFC growth showed a decline in postoperative growth of the OFC. The decline stabilizes after 2 years postoperative, resulting in a smaller OFC compared to the normal population. Second, patients with papilledema have a smaller OFC 2 years after surgery (T2) and at last follow-up (T3) compared to patients without papilledema. This finding seems to apply to FBR, ESC, and SAC. Third, a postoperative decline in the first 2 years after surgery up to a value of 0.25 SD seems to be acceptable in this population. Additionally, an OFC value at last follow-up of less than 0.5 SD is associated with the development of papilledema.

The OFC growth curve trajectory has shown to be a reliable indicator for ICV and therefore, a decline in OFC can predict the onset of ICH in patients with craniosynostosis [7, 13, 14]. Prior studies on operated sagittal synostosis patients have shown a decline in OFC and a small ICV to be associated with the occurrence of papilledema and headache, both indirect signs of ICH [1, 3, 6, 15, 16]. This is in line with our study, in which a smaller OFC is related to the occurrence of papilledema. This difference in OFC between patients with and without papilledema was seen at T2, 2 years after surgery, and continues until the last follow-up (T3). Although the 3 types of surgery result in different growth patterns, the risk of papilledema with reduced skull growth seems to concern all operated patients, independent of type of surgery. These findings support the use of OFC measurements postoperatively to detect sagittal synostosis patients at risk for ICH.

The decline in postoperative OFC seems to be a common phenomenon in sagittal synostosis patients among different types of surgery. Fearon et al. [8] described a decline in postoperative growth of the OFC in patients with sagittal synostosis who underwent occipital remodeling. Similar decline was found after complete remodeling [9, 17, 18] ESC, SAC, and endoscopic suturectomy [1, 19]. However, the progression in postoperative decline does differ between types of surgery. Our study shows that a smaller decline in OFC is found in patients who underwent a FBR. This is consistent with non-significant findings from Isaac et al. [19] The less progressive decline in OFC might be due to older age. Bergquist et al. [18] reported a smaller decline in patients who underwent cranial vault after the age of 6 months compared to patients younger than the age of 6 months. Therefore, the timing of surgery might be another explanation for the progressive decline.

In contrast, skull growth curves of trigonocephaly patients and unicoronal synostosis patients do not show the typical attenuation seen after sagittal synostosis surgery. A relation with papilledema was found in patients with trigonocephaly, but was absent in patients with unicoronal synostosis [7, 20].

In this cohort, the incidence of papilledema was 6.13%; similar results were found in the literature [1, 3–5]. Borderline or increased ICP was confirmed in 8 out of the 10 patients with papilledema. Literature indicates a sensitivity and specificity of papilledema for detecting ICH of 14–40% [21–23] and 98% [21], respectively.

Swanson et al. [24] found borderline ICP to be associated with retinal abnormalities on OCT and suggested that borderline elevated ICP might represent pathophysiology.

In this study, papilledema disappeared without intervention in a few patients. Resolved papilledema might be explained by the low sensitivity of fundoscopy, or more speculatively by a temporary skull growth arrest or reduced brain growth.

This study has four limitations that need to be taken into account. First, multiple variable testing for the correlation between papilledema and OFC could not be assessed in this cohort due to the small number of patients with papilledema. Possible confounding factors may have affected the results. To correct for possible confounding factors, a regression model should be applied. Unfortunately, due to the small number of patients with papilledema, a regression model will be overfitted and can therefore not be performed. Since our results cannot be corrected for possible confounders, one must be aware when interpreting the results. Also, a linear regression between various operation techniques could not be performed as the SAC group only comprised one patient with papilledema. Second, we used papilledema as an indicator for ICH. Although fundoscopy is a practical non-invasive and clinically relevant tool for detecting ICH in patients with craniosynostosis, it is an indirect and subjective marker of ICH. Therefore, the diagnosis of ICH should be made together with other signs of ICH, such as radiologic findings and frequent headache.

Third, due to a shift in protocol over time, three different surgical techniques are included in this study. This might have a confounding effect on the outcome. It is of added value for future studies to analyze the relation of impaired skull growth to the development of papilledema for each surgical technique separately. Unfortunately, this is was not possible in our study due to limited patients with papilledema in each group. However, our results (Table 4) indicate that there is no difference in OFC at the different time points between these surgical techniques and therefore, confounding seems to be limited. In future studies, it is recommended to analyze the relation between OFC and papilledema in a larger cohort to be able to correct for the different surgical techniques.

Fourth, there is a different length of follow-up. As FBR has been used in practice the longest, the age at which the last OFC is measured is higher compared to ESC and SAC. The last measured OFC and fundoscopy differ between patients. This led to different time points and different lengths of follow-up at long-term growth (T2T3). However, we observed that 2 years postoperatively, the OFC will continue to grow according to the predicted growth curve.

## Conclusion

This study has shown that smaller OFC is correlated to the occurrence of papilledema in sagittal synostosis patients who underwent either FBR, ESC or SAC. A decline in OFC post-surgery is common and is acceptable up to a OFC value of 0.25 SD. The risk of papilledema increases when the OFC value declines under the 0.5 SD at last follow-up.
